# Geospatial Determinants of Food Pantry Access in the Mid-Ohio Farmacy Program

**DOI:** 10.5888/pcd20.230155

**Published:** 2023-12-14

**Authors:** John Lowrey, Danielle Maestas, Thomas Beaulieu, Amy Headings, Ayaz Hyder

**Affiliations:** 1 D’Amore-McKim School of Business, Northeastern University, Boston, Massachusetts; 2 Bouvé College of Health Sciences, Northeastern University, Boston, Massachusetts; 3 Mid-Ohio Food Collective, Grove City, Ohio; 4 College of Public Health, The Ohio State University, Columbus, Ohio

**Figure:**
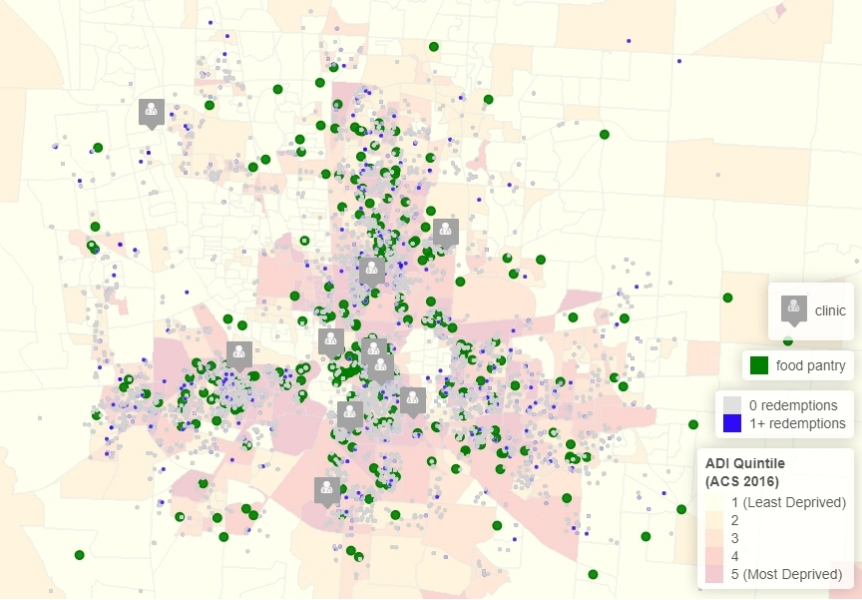
Food pantry access of 3,500 food-insecure patients in the Mid-Ohio Farmacy program, according to the Area Deprivation Index (ADI). The ADI is a residence–census block group measure of the neighborhood environment estimated from 17 variables available from the American Community Survey 5-year estimates, with yearend 2016.

## Background

Clinic-based food referral programs (FRPs) coordinate clinical and nonclinical aspects of care via clinic–community linkages. Health care providers screen patients for food insecurity and, if positive, refer them to community-based organizations (eg, food pantries). However, many patients do not redeem the referral. In a multi-institution study, 48% to 60% of referred patients did not make use of the food resource over a 6-month period (1). Although proximity to community resources encourages participation (2), there are ample barriers, which range from perceived ineligibility, stigma, lack of information retention, and inaccessibility (3). Patients’ lack of access to transportation or unavailability of public transit are 2 components of pantry inaccessibility (4–7). To better understand barriers to use, we explored the geospatial determinants of food pantry access, as part of a clinic-based FRP, using US census– and patient-level data.

A redemption is relevant because it indicates a link to the community resource, which is a precondition for health impact. Program evaluation studies report linkage rates, or the proportion of referred patients that make use of the referral at least once, and it is not uncommon to have a linkage rate below 5% (3). Low linkage rates mean that the proportion of patients that make use of the community resource with high frequency (eg, >12 visits) is also low or close to 10% to 15% (1). Low linkage rates and a low number of high-frequency users has stalled the development of clinic-based FRPs (8). The purpose of this study is to provide a geospatial perspective of linkages and explore how neighborhood environment, walkability, and pantry proximity are associated with food pantry access.

## Data and Methods

The Mid-Ohio Farmacy is a clinic-based FRP that links food insecure patients in the greater Columbus, Ohio, area to a network of more than 650 food pantries affiliated with the Mid-Ohio Food Collective (MOFC). Patients were referred to the Farmacy by 10 primary care clinics, designated as federally qualified health centers (FQHCs). At the food pantry visit, a patient received enough food for their family for approximately 2 meals per day for 10 days. Food categories ranged from fresh produce to shelf-stable goods. Patients diagnosed with a chronic metabolic condition, including obesity, hypertension, or diabetes, were screened for food insecurity at the clinic and referred to MOFC-affiliated food pantries. Income eligibility requirements to receive food from the pantry were set at below 185% of the federal poverty level (FPL). Ninety-nine percent of the FQHC patient population were at or below 200% of the FPL. For purposes of the Farmacy program, all referred patients were food pantry–eligible.

A total of 27,661 patients were screened for food insecurity at the FQHC during the study period; 43.2% were non-Hispanic Black, 25.5% were aged 60 years or older, and 63.3% were female (Table). Of all patients screened, 8,533 were identified as food insecure and referred to the Farmacy program. Redemptions were evaluated using data from PantryTrak (https://secure.pantrytrak.com) and provided by MOFC. All patient and clinic location data were retrieved from the FQHC electronic health record. FQHC clinic visits and food pantry visits spanned June 2016 to July 2020, and data were accessed on September 1, 2020. Any potential change in pantry access that resulted from the COVID-19 pandemic was averaged over the wider data set. The study protocol was approved by the institutional review board at The Ohio State University.

**Table:** Sociodemographic and Geospatial Determinants of Food Pantry Access for FQHC Patients Diagnosed with a Chronic Metabolic Condition, Mid-Ohio Farmacy Program, Franklin County, Ohio, 2016–2020

Determinant	All patients (N = 27,661)	Food-secure patients (not referred; n = 19,128)	Food-insecure patients (referred; n = 8,533)	
			0 Redemptions (n = 7,079)	≥1 Redemption (n = 1,454)
Average number of annual clinic visits per person, mean (SD)^a^	4.10 (3.98)	3.92 (3.96)	4.15 (3.58)	6.12 (5.27)
**Demographics, no. (%)**				
Non-Hispanic Black or African American	11,944 (43.2)	7,838 (41.0)	3,360 (47.5)	746 (51.3)
Non-Hispanic White	6,338 (22.9)	4,293 (22.4)	1,728 (24.4)	317 (21.8)
Age ≥60 y	7,063 (25.5)	4,304 (22.5)	2,352 (33.2)	407 (28.0)
Female	17,498 (63.3)	11,689 (61.1)	4,817 (68.0)	992 (68.2)
**Sociodemographic characteristic at block group level^b^**				
Total population, mean	1,630	1,663	1,567	1,525
Average median annual household income, $	41,956	42,659	40,693	38,816
Average median home value, $	109,573	110,912	106,846	105,083
Adult population (age ≥18 y) with high school diploma, %	83	83	82	82
Population that does not have a vehicle, %	14	13	15	16
**ADI quintile, no. (%)^c^**				
1 (Least deprived)	1,478 (5.3)	1,082 (5.7)	341 (4.8)	55 (3.8)
2	1,607 (5.8)	1,158 (6.1)	380 (5.4)	69 (4.7)
3	2,989 (10.8)	2,083 (10.9)	762 (10.8)	144 (9.9)
4	6,173 (22.3)	4,183 (21.9)	1,649 (23.3)	341 (23.5)
5 (Most deprived)	9,637 (34.8)	6,490 (33.9)	2,577 (36.4)	570 (39.2)
Missing	5,777 (20.9)	4,132 (21.6)	1,370 (19.4)	275 (18.9)
**Geographic measures, mean (SD)^d^**				
Walkscore, mean (SD)^e^	41.2 (22.1)	40.9 (22.0)	41.9 (22.4)	42.6 (21.9)
Driving distance in miles to nearest food pantry, mean (SD)	8.5 (35.8)	8.6 (39.8)	7.5 (12.7)	7.0 (26.4)
Driving duration in minutes to nearest food pantry, mean (SD)	13.7 (32.2)	13.7 (35.7)	12.8 (12.5)	12.1 (23.8)

Abbreviations: ACS 2016, American Community Survey 5-year estimates with yearend 2016; ADI, Area Deprivation Index; FQHC, federally qualified health center; API, application programming interface.

a Annual clinic visits per person and demographic data retrieved from the electronic medical record and provided by FQHCs (2).

b Sociodemographic data, including total population, annual household income, median home value, population with high school diploma, and vehicle ownership, are summarized at the US census block group–level and retrieved from ACS 2016 (3).

c ADI was the authors’ calculations based on 17 variables from ACS 2016. ADI was not defined for all patients due to missing home address information in the electronic health record. 80% (99%) of the FQHC patient population was at or below 100% (200%) of the federal poverty level. It is likely that some small proportion of patients was homeless and possible that the value of 1 of the 17 variables from ACS 2016 was missing for the patients’ block group, resulting in a missing ADI score (4).

d Geographic measures were calculated using coordinate locations (latitude, longitude) for patients’ randomized home address.

e WalkScore, a point-specific measure of mobility that accounts for variation in built environment within a neighborhood block group (eg, major highways, sidewalks, urban green spaces), was retrieved using the WalkScore API. Driving distance and duration were retrieved using the Distance Matrix API from Google Maps with the patients’ home address and the nearest food pantry location as the origin and destination, respectively.

Farmacy redemptions were evaluated according to the patient’s home address and food pantry and clinic locations. Street-level home address information was geocoded to a latitude and longitude location and randomized with an offset of 500 meters to protect patient health information. This randomized location was used as the basis to evaluate geospatial determinants of access, including transportation and mobility-related factors.

To approximate the neighborhood environment, we used the Area Deprivation Index (ADI), a residence–census block group measure of neighborhood environment used extensively in the health sciences literature (9–11). ADI scores were estimated according to the literature (12). For each patient, the randomized home address was mapped to a census block group using a nesting function and the TIGER/Line Shapefiles for the state of Ohio. The ADI is based on data from the US Census Bureau 2016 American Community Survey (ACS), including 17 variables that span the domains of income, education, household size, housing quality, and affordability. The 17 variables come from 60 months of collected data and represent a 5-year average from January 1, 2012, to December 31, 2016. Higher ADI scores indicate greater neighborhood deprivation.

To approximate mobility, we used the randomized latitude and longitude point location for a patient’s home address and the WalkScore Application Programming Interface (API) (www.walkscore.com). WalkScore is a point-specific measure of mobility, which accounts for variation in built environment within a neighborhood block group, such as major highways, sidewalks, and urban green spaces. WalkScore has been used in other studies (13), but it is usually estimated using zip code centroids, which is a less precise measure of mobility relative to a patient’s point location.

We also examined the driving distance (miles) and duration (minutes) between a patient’s home address and food pantry using the Google Maps Distance Matrix API. The inputs were the latitude and longitude coordinate locations for a trip, with the origin and destination of the patient’s home address and nearest food pantry, respectively. Although patients vary in how they select a food pantry, in part due to unobserved preferences, the distance to the nearest pantry represents a consistent and comparative measure of access. We could not use the first or most visited food pantry since 80% of referred patients had no pantry visits or 0 redemptions. The nearest food pantry was the one with the smallest great-circle-distance from a patient’s home address.

## Highlights

We present several noteworthy findings. First, the linkage rate was 17%, or 1,454 patients with 1 or more food pantry visits of 8,533 patients referred. Among patients with 1 or more redemptions, the distribution of food pantry visits presented a strong positive skew; 53% (n = 765) of patients had 1 to 3 visits to the food pantry. Second, spatial point patterns and trends suggested that food pantry locations are dense, across both inner-city regions and the surrounding suburbs of the study area. Regionally consistent clustering patterns could partially explain low variation in driving distances to the nearest food pantry across the 2 main Farmacy redemption cohorts.

Third, since patients are referred to a network of existing food pantries with significant clustering, the average distance (in miles) to the nearest pantry did not appear to be associated with redemption. The average distance to the nearest pantry for patients who had 1 or more redemptions was 7.0 miles, and the average distance for patients who had no redemptions was 7.5 miles; the difference between the 2 was not significant (*t* =1.18, *P* = .23) (Table). The difference in mean driving duration (in minutes) was also not significant (*t* =1.43, *P* = .15). WalkScore did not vary across the 2 redemption status cohorts. Geospatial factors related to food pantry proximity or mobility did not strongly influence food pantry access, which contrasts with existing findings on the importance of proximity (2).

Fourth, the proportion of referred patients that lived in the most deprived areas, with an ADI score in quintile 5, ranged from 36.4% in the group with 0 redemptions to 39.2% in the group with 1 or more redemptions (Table). We focused on the highest quintile because it is consistently identified as a risk factor for health (10,11). The difference in proportions was significant (${\hat{p}}_{1}$–${\hat{p}}_{2}$ = 0.03, *P* = .03), although the magnitude of the difference was small (close to 3%). Neighborhood environment, as measured by ADI, may potentially explain differences in food pantry access in a clinic-based FRP.

## Action

Adults with chronic metabolic conditions that live in a deprived neighborhood may be more likely to redeem their referral and visit a food pantry. Understanding what motivates patients to visit the food pantry in clinic-based FRPs will help expand the use of these programs in practice and address hunger as a risk factor for obesity (14). Future studies of clinic-based FRPs should be mindful of how geospatial components of access can influence linkage. Concurrent interventions that address pantry access could also resolve socioeconomic barriers to use, such as perceived ineligibility or stigma.

The main limitation of this study was the lack of patient- and trip-specific data. For example, we did not capture all patient trips, vehicle ownership or availability, or mode of transit. These data are relevant because a patient could visit a food pantry as part of a trip with multiple locations. In other words, the trip does not have to originate from the patients’ home address — a patient could stop by the pantry on their way home from work. Mode of transit could explain differences in food pantry access since walking, use of public transit, or driving a personal vehicle have different levels of convenience and cost.

Another possible limitation results from our use of a randomized offset for the patients’ home address. Namely, this offset could result in a misclassification of the block group, which is especially true for a patient who lives near a contiguous boundary. A more standardized approach would be to map a patient to their exact block group — or the smallest unit of US census geography permitted — and use the centroid for network distance calculations. Furthermore, ADI is based on a 5-year average of ACS data, so our measure of ADI did not capture annual changes in geospatial access patterns.
